# Pleural Infection Caused by Nocardia farcinica: Two Cases and Review of the Literature

**DOI:** 10.7759/cureus.14697

**Published:** 2021-04-26

**Authors:** Graciella Bagüeste, Jose M Porcel

**Affiliations:** 1 Internal Medicine, Arnau de Vilanova University Hospital, Lleida, ESP

**Keywords:** nocardia farcinica, empyema, pleural effusion, antibiotics

## Abstract

*Nocardia farcinica* is a rare Nocardia species causing localized (lung, brain, skin) and disseminated infections. Predisposing factors include the chronic use of corticosteroids, organ transplantation and other immunocompromise conditions. Pleural empyema caused by this microorganism has scantily been reported. We describe two cases of pleural infection by *N. farcinica* that occurred in patients with a kidney transplant and cirrhosis, respectively. The first patient died soon after hospitalization, while the second survived nocardiosis (despite having significant adverse events to antibiotics) but eventually succumbed to other infectious complications. In this infectious disease, in which the duration of therapy is typically long and pleural space drainage is frequently required, bacterial susceptibility to antimicrobial agents should be tested.

## Introduction

*Nocardia farcinica* is a particularly virulent Nocardia species that causes both localized and disseminated infections, mostly in the setting of immunocompromised conditions (e.g., glucocorticoids, calcineurin inhibitors and other immunosuppressive medications, hematologic and solid-organ transplant recipients, malignancy, HIV disease, diabetes). The infection is mainly acquired by inhalation, less commonly by direct inoculation through the skin, and frequently results in disseminated disease. In a Spanish series of 1,119 strains of the Nocardia genus, *N. farcinica* represented 11.4% of the isolates [1]. About 60% of *N. farcinica* strains were isolated from bronchial secretions, but only 4% from lung/pleural fluid samples [1].

Even though pneumonia is the most common manifestation of *N. farcinica*, pleural involvement is infrequent, with only a few cases being reported [2-14]. This article describes two additional patients with pleural empyema by this gram-positive aerobic actinomycetes and succinctly reviews the literature on the subject.

## Case presentation

Case 1

**Figure 1:**
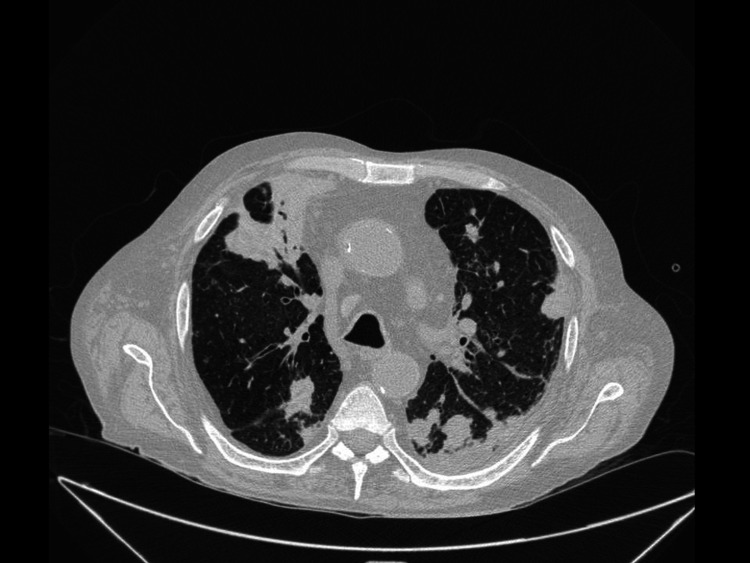
Chest CT scan showing pulmonary nodules and a left-sided pleural effusion

A 73-year-old man was hospitalized for a two-week history of dyspnea. He had undergone a kidney transplant two years earlier and was taking prednisone (5 mg/d), tacrolimus (2.5 mg/d) and mycophenolate mofetil (1 g/d). A chest X-ray and CT showed multiple pulmonary nodules, some of which were cavitated, along with consolidations and a left free-flowing pleural effusion occupying about 25% of the hemithorax (Figure 1). A diagnostic thoracentesis displayed a non-purulent exudate with the following characteristics: erythrocyte count 38,500 cells/µL, leukocytes 987 cells/µL (80% neutrophils), lactate dehydrogenase 737 U/L, glucose 95.5 mg/dL, adenosine deaminase 14.4 U/L, pH 7.30 and C-reactive protein 133 mg/L. Empiric antibiotic therapy with ceftriaxone and trimethoprim-sulfamethoxazole (TMP-SMX) was initiated. Subsequently, ceftriaxone was replaced by piperacillin-tazobactam. *N. farcinica* was isolated from the pleural fluid and blood cultures after eight days of incubation. The antibiogram showed that it was susceptible to TMP-SMX, linezolid, amikacin, imipenem and amoxicillin-clavulanate. Also, an active cytomegalovirus (CMV) infection was diagnosed based on the presence of CMV replication in the blood (240,000 copies/mL). Tacrolimus was discontinued because of its high blood levels (13.35 ng/mL). The patient died six days after hospital admission before any of the previous microbiological results became available. The autopsy confirmed the presence of bilateral pulmonary and pleural nocardiosis, as well as CMV pneumonitis; both entities probably contributing to death.

Case 2

A 49-year-old woman with a history of alcoholic liver cirrhosis (Child Pugh C and MELD score 19) was admitted to the hospital because of fever, dyspnea and right pleuritic chest pain of one-week duration. In some areas of ground-glass opacification, a large loculated right-sided pleural effusion and ascites were seen on the chest CT. A diagnostic pleural tap showed a non-purulent exudate with the following characteristics: erythrocyte count 34,900 cells/µL, leukocytes 2,411 cells/µL (58% neutrophils), lactate dehydrogenase 595 U/L, glucose 63.1 mg/dL, adenosine deaminase 17.6 U/L, pH 7.43 and C-reactive protein 52 mg/L. The analysis of peritoneal fluid was consistent with portal hypertension (serum-ascites albumin gradient >1.1 g/dL). Empirical antibiotic therapy with cefotaxime was started along with two serial therapeutic thoracenteses of 600 mL each. *N. farcinica* grew on pleural fluid cultures after 6 days of incubation, but an antibiogram was not done. Blood cultures were negative. Cefotaxime was replaced by linezolid 600 mg po q12h. One month later, the patient developed myelosuppression secondary to linezolid, which had to be withdrawn. At that time, there remained a small pleural effusion. The patient needed colony-stimulating factors (filgrastim), platelet and red cell transfusion. Ciprofloxacin (500 mg po q12 h) was initiated. After three months with the new antibiotic regimen, the patient suffered a cardiorespiratory arrest due to an acquired long QT syndrome that was attributed to fluoroquinolones. A long ICU stay (two months) was needed during which meropenem, vancomycin and fluconazole were administered for a number of infectious complications and cirrhosis decompensation. At hospital discharge, no further antibiotics were prescribed. One year later, the patient required a total right hip arthroplasty and died of septic shock secondary to an early-onset prosthetic hip infection.

## Discussion

Pleural infection by *N. farcinica* is rare, with about 16 cases previously reported in the literature (Table 1), according to a PubMed search (keywords: [pleural or empyema or pleuritis] and Nocardia farcinica; time period from inception to March 2021) [2-14]. The general series of nocardiosis briefly mention a few additional cases of pleural involvement by* N. farcinica*, but with information so incomplete that they cannot be incorporated into this review. 

**Table 1:** Previous cases of pleural empyema by N. farcinica reported in the literature AG, aminoglycoside; AMK, amikacin; Amox-clav, amoxicillin-clavulanate; Amp-sulb, ampicillin-sulbactam; BAL, bronchoalveolar lavage; CIP, ciprofloxacin; CLL, chronic lymphocytic leukemia; CIP, ciprofloxacin; F, female; HIV, human immunodeficiency virus; IMP, imipenem-cilastatin; ITP, immune thrombocytopenic purpura; Levo, levofloxacin; M, male; MER, meropenem; Mino, minocycline; NA, not available; SMX, sulfamethoxazole; TMP-SMX, trimethoprim/sulfamethoxazole

Reference	Age	Sex	Predisposing factors	Site of Infection	Diagnostic samples	Therapy	Death due to nocardiosis
Nakajima et al. 1999 [2]	21	F	Systemic lupus erythematosus; corticosteroids	Pleura	Pleural fluid	Pleural drainage + intrapleural IMP + Mino + TMP-SMX	No
Torres et al. 2000 [3]	NA	NA	CLL	Lung, pleura	Pleural fluid	NA	NA
Torres et al. 2000 [3]	70	M	None	Lung, pleura	Pleural fluid, sputum	SMX	No
Torres et al. 2000 [3]	44	M	None	Lung, pleura, brain	Pleural fluid	Pleural drainage + IMP + CIP + AG	Yes
Ando et al. 2001 [4]	69	F	ITP; corticosteroids	Pleura	Pleural fluid	Therapeutic thoracentesis + IMP + TMP-SMX + Mino	No
Arunthathi et al. [5]	NA	M	Corticosteroids, thalidomide	Pleura	Pleural fluid	AMK + Mino	NA
Severo et al. 2005 [6]	75	M	Corticosteroids	Lung, pleura, thyroid, heart, kidneys, brain, bone, lumbosacral soft tissue	Blood, thyroid, sputum	TMP-SMX	Yes
Rivero et al. 2008 [7]	42	M	Heart transplantation; corticosteroids, cyclosporine, and mycophenolate mofetil	Pleura, pericardium, brain	Pleural fluid, pericardial fluid	Pleural and pericardial drainages + TMP-SMX + IMP + AMK + linezolid	No
Parande et al. 2010 [8]	27	M	HIV	Lung, pleura	Pleural fluid, sputum	Pleural drainage + TMP-SMX + AMK	Yes
Budzik et al. 2012 [9]	78	M	Intraarticular corticosteroids	Knee joint, lung, pleura	Synovial fluid, blood, lung	TMP-SMX	Yes
Ishiguro et al. 2017 [10]	82	M	Diabetes mellitus	Pleura, lung, knee	Pleural fluid, blood, synovial fluid	Pleural and joint drainages + Amp-sulb + Mino + IMP + Levo	No
Canouï et al. 2017 [11]	30	M	Hematopoietic stem cell transplantation; corticosteroids, chemotherapy, rituximab	Pleura, lung	Pleural fluid, pleural biopsy, BAL	Pleural drainage + MER + AMK + doxycycline	No
Huang et al. 2019 [12]	56	M	NA	Pleura, lung	Pleural fluid	NA	NA
Huang et al. 2019 [12]	76	M	NA	Pleura, lung	Pleural fluid	NA	NA
Nasri et al. 2019 [13]	91	M	Astrocytoma	Meninges, lung, pleura	Cerebrospinal fluid	TMP-SMX + IMP	Yes
Zayet et al. 2020 [14]	68	M	Corticosteroids	Pleura, lung, brain	Pleural fluid, BAL	Pleural drainage + Amox-clav + IMP + TMP-SMX	No

Nocardiosis mostly affects patients with impaired cell-mediated immunity, as exemplified by these two new observations where the infection developed in the context of a renal transplant recipient under immunosuppressive therapy and advanced cirrhosis, respectively. In a retrospective compilation of 53 patients with *N. farcinica* infections up to the year 1999, 85% had predisposing factors, among which the most frequent was the chronic use of corticosteroids [3]. In fact, at least half of the patients with a nocardial pleural infection listed in Table 1 had a history of corticosteroid treatment.

Pneumonia, brain abscesses and skin infections are the major clinical presentations of *N. farcinica* infection, though in nearly one-third of the cases the disease disseminates, particularly to the central nervous system [3]. Other infections, such as aspergillosis, CMV disease (Case 1) and gram-negative bacteria, may occur concomitantly to nocardiosis [15]. In pulmonary nocardiosis, findings on chest imaging may be variable and include solitary or multiple nodules (Case 1), as well as multifocal consolidations or ground-glass opacities (Cases 1 and 2). Cavitation is usually restricted to immunocompromised patients [15]. Sometimes, the disease may initially resemble tuberculosis, particularly if upper lung lobes are involved and weakly acid-fast filaments are seen on respiratory samples. Nocardia filaments may or may not be acid-fast. Although Nocardia may grow in most routine bacterial, fungal and mycobacterial media, the laboratory should be notified of this possibility in order to use selective media (e.g., modified Thayer-Martin, Columbia blood agar). In one review series, the median time required for the isolation of *N. farcinica* in various samples was four days, but growth may take several weeks [3]. If available, molecular diagnostics (e.g., 16S RNA gene sequencing) may allow the identification and speciation of Nocardia isolates.

Two-thirds of pleural infections by *N. farcinica* reported in the literature were managed with tube thoracostomy or therapeutic thoracentesis, in addition to antibiotics (Table 1), as is generally indicated in patients with positive pleural fluid cultures [16]. Antibiotic susceptibility testing is mandatory because treatment should be based on it. According to large series, *N. farcinica* is uniformly susceptible to linezolid and amikacin, commonly susceptible to imipenem and amoxicillin-clavulanate, variably susceptible to TMP-SMX, usually resistant to ciprofloxacin, and typically resistant to third-generation cephalosporins, minocycline and aminoglycosides other than amikacin (Table 2) [1,17-19].

**Table 2:** Antimicrobial resistance (non-susceptible isolates) of Nocardia farcinica according to different series (n>100) Amox-clav, amoxicillin-clavulanate; AMK, amikacin; CIP, ciprofloxacin; IMP, imipenem-cilastatin; Mino, minocycline; USA, United States of America; TMP-SMX, trimethoprim/sulfamethoxazole

Country [ref.]	No. of isolates	TMP-SMX	IMP	AMK	Linezolid	Mino	Amox-clav	Cefotaxime	Ceftriaxone	CIP
Spain [1]	128	45.3%	3.9%	1.6%	3.1%	89.1%	18%	54.7%	-	48.4%
France [17]	149	4%	23%	1.4%	0%	12.8%	20.1%	79.7%	80.5%	41.9%
USA [18]	105	80%	25%	0%	0%	79%	10%		93%	72%
USA [19]	319	1%	17%	0%	0%	93%	4%	-	97%	51%

Suggested initial regimens for pleuropulmonary nocardiosis usually include the combination of TMP-SMX (15 mg/Kg/day of the TMP component IV/po divided in 2-4 doses) plus imipenem (500 mg IV q6h), with the option to add amikacin (7.5 mg/Kg IV q12 h) in severe infections [20]. After 3-4 weeks of intravenous therapy and documented clinical improvement, patients may be switched to oral monotherapy. Duration of antibiotic treatment is generally long (6-12 months). Although these guidelines apply to nocardiosis in general, once the Nocardia species is identified and susceptibility testing results are available, the antibiotic regimen must be adapted. We suggest the combination of imipenem and amikacin for the induction therapy of *N. farcinica* infections. Although linezolid seems an attractive option, its use for more than a few weeks is normally precluded by the risk of hematologic toxicity (Case 2) and neurotoxicity. The mortality of *N. farcinica* pleural infections is not negligible, with more than one-third of fatalities occurring among the reviewed patients (Table 1). To reduce mortality, an early diagnosis and prompt initiation of adequate antibiotic treatment are imperative.

## Conclusions

Pleural infection by *N. farcinica* is rare, with only 16 previously reported cases prior to the new ones exposed herein. This condition should be suspected in immunocompromised subjects with pleural effusions and pulmonary nodules/consolidations. Pleural fluid is an optimal specimen for the isolation of the microorganism. In patients with severe disease or immunocompromise, combination empirical therapy (≥2 drugs) is initially warranted. In particular, consideration should be given to the use of amikacin, imipenem and linezolid.
